# Sexual motivations for engaging in chemsex behaviours

**DOI:** 10.1192/j.eurpsy.2023.1378

**Published:** 2023-07-19

**Authors:** J. Curto Ramos, M. T. Heredia Soriano, I. Azqueta, I. De Ema Lopez, R. Molina Prado

**Affiliations:** 1 Department of Psychiatry, Clinical Psychology and Mental Health, La Paz University Hospital, Madrid, Spain, Hospital la Paz, Madrid; 2 ONG APOYO POSITIVO, MALAGA; 3 ONG APOYO POSITIVO; 4 CAD TETUÁN. Instituto Adicciones Madrid Salud; 5 CAD ARGANZUELA. Instituto Adicciones Madrid Salud, Madrid, Spain

## Abstract

**Introduction:**

The intentional use of drugs before or during sexual intercourse (chemsex) is a phenomenon of special importance in the MSM (men who have sex with men) population due to its impact on mental, physical and sexual health. Sexual health issues related to chemsex practice have been described such as difficulties in achieving sober sex, erectile dysfunction or problems with sexual desire.**Objectives:** The objective of this study was to understand the sexual motivations for chemsex practice o a group participantes of a sexual health program for chemsex users in two Drug Substace Use Disorder Clinics in Madrid.

**Methods:**

Qualitative research approach. We analyze an anonymous survey with chemsex users with open answer questions about the motivations for chemsex practice. Data analysis was based on thematic analysis of content.

**Results:**

Different qualitative studies have examined the motivations for engaging in chemsex. The participants identified two main raisons: pleasure and losing inhibitions. We analyed the inhibitions described by participants: difficulties with arousal, ejaculation, social interaction in sexual context, difficulties in situations that require intimacy, sexual practices that make them feel guilt/shame (for example BDSM) problems with “erotic” self-esteem: rejection of non-normative bodies or towards non- normative gender expression perceived as undesirable.

**Conclusions:**

Understanding the sexual motivations for engaging in chemsex seems necessary to develop and multidisciplinary approach. Mental health proffesionals should consider sexual counselling and sexual therapy for chemsex users in their treatment.

**Disclosure of Interest:**

None Declared

